# Step-Gap in Upward Support: The Role of Biological Relatedness and Childhood Co-residence Duration

**DOI:** 10.1093/geronb/gbad179

**Published:** 2023-12-21

**Authors:** Hans Hämäläinen, Antti O Tanskanen, Jenni Pettay, Mirkka Danielsbacka

**Affiliations:** INVEST Research Flagship Centre, University of Turku, Turku, Finland; Department of Social Research, University of Turku, Turku, Finland; Department of Social Research, University of Turku, Turku, Finland; Population Research Institute, Väestöliitto, Helsinki, Finland; INVEST Research Flagship Centre, University of Turku, Turku, Finland; Department of Social Research, University of Turku, Turku, Finland; INVEST Research Flagship Centre, University of Turku, Turku, Finland; Population Research Institute, Väestöliitto, Helsinki, Finland; (Social Sciences Section)

**Keywords:** Biological children, Biological parents, Intergenerational help, Stepchildren, Stepparents

## Abstract

**Objectives:**

Although prior research has detected a step-gap in intergenerational relationships in various aspects, there is a lack of studies examining adult children’s support toward their biological parents and stepparents. We investigated (i) whether adult children provide more support to their biological parents than stepparents and (ii) whether the childhood co-residence duration is associated with the support given to stepparents.

**Methods:**

The data were drawn from the German Family Panel (pairfam). Upward support was indicated by using 3 different measures, namely, financial, practical, and emotional support provided by adult children to their biological parents and stepparents. A path analysis was conducted to detect the potential differences regarding upward support.

**Results:**

More support was channeled toward the biological parents than the stepparents. Moreover, the length of co-residence during childhood and adolescence was positively associated with the frequency of support provided toward the stepparents. Consequently, an increased childhood co-residence duration decreased the step-gap in upward support, although it did not fully eliminate it.

**Discussion:**

The findings showed that stepparents are in a more disadvantaged position than the biological parents regarding receiving support from their adult children. In the context of a high old-age dependency ratio, it is important to recognize that stepparents may not have the opportunity to receive adequate support from their families as compared to individuals with biological children.

Owing to the rising divorce rates and repartnering, the number of stepfamilies (i.e., the families co-residing with underage stepchildren) in Western countries has increased substantially (Thomson, 2014). Currently, stepfamilies are estimated to form approximately 10% of all families in Europe and about 14% in Germany (Steinbach et al., 2016). In stepfamilies, a new adult becomes a part of the family by forming a relationship with the stepchild’s biological parent without necessarily choosing the child in question (Arránz Becker et al., 2013). This is argued to lead to a weaker parent–child relationship quality in such families compared to intact families (e.g., Steinbach & Hank, 2016).

As the Western populations are aging rapidly, and the old-age dependency ratio is worsening (Organisation for Economic Co-operation and Development [OECD], 2023), the question of upward support in stepfamilies is a burning topic (e.g., Patterson et al., 2022). When parents age, receiving support outside their household becomes typically more important for their daily lives, owing to their declining functional abilities. Previous studies have shown that support is widely provided among family members, and in addition to their partners, adult children are the most significant source of informal support for older adults (e.g., Silverstein & Giarrusso, 2010). However, it is unclear whether this also considers stepchildren. Although prior research has found that stepparent–stepchild relationships tend to suffer from poor relationship quality (e.g., Arránz Becker et al., 2013; Steinbach & Hank, 2016), insufficient studies have examined the provision of support to the stepparents by their stepchildren (but see Pezzin et al., 2008).

## Step-Gap in Intergenerational Relationships

The term “step-gap” is often used to describe the differences in the relationship quality between the step-relatives and their biological counterparts (e.g., Arránz Becker et al., 2013; DeLongis & Preece, 2002). The existence of the step-gap has been explained by theoretical perspectives focusing on variations in biological relatedness (“biological relatedness effect”) and the influence of social norms (“social norms effect”). From the perspective of biological relatedness, individuals typically exhibit a strong tendency to favor family members with whom they have genetic ties over those who are not biologically related (Tanskanen & Danielsbacka, 2019). Children obviously have a genetic link with their biological parents but not with their stepparents. The presence of this biological connection has led some family researchers to suggest that adult children tend to be more devoted to their biological parents than to their stepparents (e.g., Cheal, 1988; Ganong & Coleman, 2006).

From the viewpoint of the social norms, the key elements regulating intergenerational support are the social expectations and obligations to support family members (Bengtson, 2001; Bengtson & Roberts, 1991). However, notable disparities might arise in the expected roles of adult children toward their biological parents versus their stepparents (Noël-Miller, 2013; Silverstein & Giarrusso, 2010). While the traditional nuclear family may operate as a “complete institution” in the sense that it poses well-defined institutionalized familial roles and mutual relationships, the stepfamily has been described as an “incomplete institution” because it lacks such established guidelines (Cherlin, 1978; Ganong & Coleman, 1997). As a result, the norms that encourage adult children to support their biological parents could be robust, while comparable norms might be weaker or even absent in stepfamilies (van Houdt et al., 2018). This may lead to diminished support toward stepparents compared to biological parents.

Prior research has detected a step-gap in intergenerational relationships in various settings. First, it has been found that *downward* support provided by parents to children is more often channeled toward biological than step-related descendants (e.g., Arat et al. 2021; Pettay et al., 2023). Second, adult children have weaker *normative obligations* to provide support to their stepparents than biological ones (i.e., respondents generally think that people should help biological parents more than stepparents; van Houdt et al., 2018). Third, there is evidence that the stepparent–stepchild *relationship quality* (measured as contact frequency and emotional closeness) tends to be poorer compared to biological parent–child relationships (e.g., Arránz Becker et al. 2013; Steinbach & Hank, 2016).

However, there is a gap in studies that specifically explore the differences in the provision of upward support to stepparents as opposed to biological parents (for reviews, see Carr & Utz, 2020; Fingerman et al., 2020; Silverstein & Giarrusso, 2010). A notable exception is the study by Pezzin and colleagues (2008), which detected that biological parents received more practical and financial support from adult children compared to stepparents. It is worth noting that this study focused solely on older parents who were both disabled and without partners, which drastically limits the generalizability of its findings. Consequently, as far as our knowledge goes, there has not been an analysis of the step-gap in upward support utilizing a broader sample of older adults.

Otherwise, a handful of prior studies have investigated whether the *family structure* of older adults is linked to the support they receive from adult children, and provided *indirect* evidence for the step-gap phenomenon. Specifically, these studies have found that older parents who have stepchildren tend to receive less care (Kalbarczyk, 2021; Patterson et al., 2022; Pezzin & Schone, 1999), less time-intensive assistance (Wiemers et al., 2019), and less practical help (Kalbarczyk, 2021) from their adult children compared to parents who only have biological children. These investigations have explored how family structure, involving biological and/or stepchildren, affects the support received by parents. However, they have not directly addressed the differences in support provided by biological children versus stepchildren.

## Childhood Co-residence Duration

It is important to recognize that family relationships are not stagnant. Previous research highlights that the time individuals spend together during childhood plays a crucial role in shaping and fortifying social bonds among them (“childhood co-residence effect”; Kalmijn, 2013; Tanskanen et al., 2021). A clear contrast in co-residence arises between children and their biological parents as opposed to children and stepparents. Biological parents are usually present from birth, whereas stepparents often become part of the family at a later point, that is, after establishing a relationship with the stepchildren’s biological parents. While biological parents typically have a significant role in the lives of their children regardless of co-residence (e.g., Tanskanen & Erola, 2017), the co-residence effect is critical in determining whether a stepparent is perceived as both a family member and a parental figure (Schmeeckle et al., 2006).

Here, we argue that the childhood co-residence duration may serve as the key factor affecting the width of the upward step-gap because it provides the basis for the formation of the stepparent–stepchild relationship (Kalmijn et al., 2019). The longer the stepparent and stepchild live in a shared household, the more opportunities they have to develop their relationship. Living with stepparents during childhood can even foster a “feeling of kinship” regardless of the biological relatedness, leading stepparents and stepchildren to perceive themselves as emotional kin (Rotkirch, 2018). Moreover, increased length of co-residence with stepparents may result in stronger normative obligations toward stepparents (van Houdt et al., 2018). This implies that as these stepchildren grow up, they could be more inclined to provide additional support to their stepparents during later stages of life.

Furthermore, the co-residence duration can be considered a proxy for the parents’ investment in their children (Kalmijn et al., 2019). It is argued that the investments received from parents earlier in life may influence the current support that their adult children provide to them through long-term reciprocity, as adult children may feel obligated to repay the investments they received earlier (Silverstein et al., 2002). Individuals have a strong tendency to invest at least some time and resources in their biological kin despite the earlier investment received from them (Hämäläinen et al., 2020). On the contrary, support among nonkin is typically more grounded in reciprocity compared to biological relationships (Rotkirch et al., 2014). Therefore, reciprocity is expected to have a significant impact on adult children’s support toward stepparents.

## Study Aims

In this study, we examine whether adult children provide differing levels of support to their stepparents compared to their biological parents. To the best of our knowledge, this is the first study to investigate the step-gap phenomenon in upward support by using an extensive population-based sample. This stands in contrast to the study by Pezzin and colleagues (2008), who explored the step-gap in upward support but focused on a very specific group of older adults, namely, disabled and unpartnered individuals. Drawing on the “biological relatedness effect,” which suggests a preference for biological kin over nonbiological relations, and the “social norms effect,” which proposes that stepfamilies lack institutionalized guidelines toward supporting stepparents, we test the following hypothesis:

*Hypothesis 1: Adult children provide more support toward biological parents than stepparents.*

Another novel contribution of our study is that we examine whether the length of childhood co-residence between stepchildren and stepparents is associated with the level of support adult children provide to their stepparents. Based on the “childhood co-residence effect,” which suggests that a longer co-residence duration strengthens stepparent–stepchild relationships and increases upward support via long-term reciprocity, we propose:

*Hypothesis 2: Longer duration of childhood co-residence is associated with higher levels of support provided by adult children to their stepparents.*

## Method

In this study, we utilized data from the German Family Panel (pairfam), which offers wide-ranging information on the intergenerational relations and socioecological factors from three birth cohorts born in 1971–1973, 1981–1983, and 1991–1993 (Brüderl et al., 2019; Huinink et al., 2011). We employed the data from the second wave, collected in 2010–2011, as the questions related to childhood living arrangements and intergenerational support were recorded in this wave. For the analyses, we included the older cohorts who were aged 27–29 and 37–39, respectively, at the time of data collection. The youngest age cohort was omitted because, at the time of data collection, they were mostly underage (or had just become adults) and, thus, still dependent on their parents.

Our dependent variables measured the financial, practical, and emotional support provided by the respondents to their parents during the 12 months preceding the survey. Financial support refers to giving money, practical help to assist with shopping, housework, or yardwork, and emotional support to whether the respondents discuss their parents’ concerns or problems with them. All three types of support were measured on a scale ranging from zero to four (0 = never, 1 = seldom, 2 = sometimes, 3 = often, 4 = very often). Respondents were asked to report the support they had provided to their biological mothers and fathers, respectively. Additionally, participants were asked whether they had provided support to their mothers’ partners (stepfathers) or fathers’ partners (stepmothers). If adult children had both stepfathers and stepmothers, the questions were limited to stepfathers only (Brüderl et al., 2019). To compare the assistance provided to biological and stepparents, “parent type” (mother, father, stepfather, and stepmother) was used as the main independent variable (*N* = 8,949).

To obtain more robust results, we controlled for various sociodemographic factors that have been identified as having associations with the provision of intergenerational support in earlier research. Prior studies have indicated that adult daughters provide more support to their parents than adult sons do, and that increased age is associated with a decreased provision of support (e.g., Hämäläinen & Tanskanen, 2021; Mulder & van der Meer, 2009). Therefore, we controlled for respondents’ gender and birth cohort (1 = 1971–1973; 2 = 1981–1983). As previous research has shown that those with greater resources have a higher probability of providing support (e.g., Brandt et al., 2009; Deindl & Brandt, 2011), the educational level of adult children was controlled. The education variable was based on the International Standard Classification of Education-97 and was treated as continuous in the analyses, ranging from lower secondary or less to tertiary education. The family structure of adult children is also associated with their provision of support; those who have a partner and children of their own are less likely to provide support to their parents than those without a partner or children (e.g., Brandt et al., 2009; Mulder & van der Meer, 2009). Consequently, we controlled for co-habitation status (0 = co-habiting with someone; 1 = not co-habiting) and parenthood status (0 = no children; 1 = at least one child). Given the potential cultural differences in the norms of familial support (e.g., Dykstra & Fokkema, 2011; Kalmijn & Saraceno, 2008), we controlled for the ethnic background based on the parents’ birth country (0 = German native; 1 = other).

Our data enabled us to also take into account important sociodemographic factors pertaining to the respondents’ parents. We used their age (ranging from 25 to 87 years) as a proxy for the need for help, acknowledging that aging individuals often experience declining health and a consequent increased reliance on family support (e.g., Deindl & Brandt, 2011; Mulder & van der Meer, 2009). Furthermore, we controlled for the parents’ co-habitation status (0 = co-habiting with someone; 1 = not co-habiting), given that those who co-habit tend to receive less support (e.g., Brandt et al. 2009; Deindl & Brandt, 2011). We also took into account the travel time from the respondents’ home to their (step)parents’ dwelling, because increased geographic distance usually decreases the level of provided support (e.g., Kalbarczyk, 2021; Mulder & van der Meer, 2009). Travel time was treated as a continuous variable, ranging from 0 (living in the same household) to 5 (takes at least 3 hours to travel). Unfortunately, data on (step)parents’ health, education, income, or the number of other (step)children were not available, so we were unable to control for these factors. Finally, prior studies considering the step-gap in intergenerational relations have indicated that differences in relationship quality can be influenced by both biological relatedness and the gender of (step)parents (e.g., Arránz Becker et al., 2013; Steinbach & Hank, 2016). Therefore, we explored the support adult children offer to mothers, fathers, stepfathers, and stepmothers separately.

To further investigate the support provided by the adult children to their stepparents (*N* = 984), two additional variables were considered. First, the duration of childhood co-residence with stepparents was determined from the questions about respondents’ living arrangements before the age of 18 years. It is assumed that the stepparent with whom the respondent lived with during childhood corresponds to the stepparent at the time of the survey. Hence, the childhood co-residence duration was calculated as 18 years minus the age at which the respondent started living with the stepparent (ranging from 0 to 18 years). Second, a variable measuring how emotionally close the respondents felt to their biological parents (i.e., closeness to the partner of stepparent) was included, as the latter can be an important relationship mediator between the stepchildren and stepparents (Klaus et al., 2012). The scale ranged from 0 (not at all) to 4 (very close). The descriptive statistics for all dependent and independent variables are presented in Tables 1 and 2, respectively.

**Table 1.** Descriptive Statistics of the Respondents

Variable	*n*	Missing %
Birth cohort		0
1981–1983	2,058	
1971–1973	2,571	
Gender		0
Male	2,042	
Female	2,587	
Ethnic background		2.21
German native	3,479	
Other	1,050	
Level of education		0.09
Lower secondary or less	381	
Upper secondary	2,091	
Postsecondary	727	
Tertiary	1,426	
Co-habitation status		1.16
Not co-habiting	1,285	
Co-habiting	3,291	
Parenthood status		0.09
No children	1,882	
At least one child	2,742	

**Table 2.** Descriptive Statistics of the Parents

Variable	Mother			Father			Stepfather			Stepmother			Missing
	*n*	Mean	*SD*	*n*	Mean	*SD*	*n*	Mean	*SD*	*n*	Mean	*SD*	%
Number of observations	4,344			3,615			681			309			
Financial help		0.28	0.71		0.19	0.61		0.08	0.4		0.06	0.39	0.20
Practical help		1.23	1.15		1.1	1.15		0.64	0.98		0.35	0.75	0.17
Emotional support		1.87	1.07		1.36	1.04		0.72	0.96		0.78	0.96	0.21
Age of the parent		58.57	7.47		61.34	7.67		56.42	8.75		55.55	9.79	9.95
Childhood co-residence duration		17.55	2.13		16.99	3.39		2.72	5.07		0.8	2.90	0.00
Parent’s co-habitation status													0.16
Not co-habiting	1,103			493			104			54			
Co-habiting	3,233			3,119			575			254			
Travel time to parent’s home													1.91
Live in the same house	423			334			34			13			
Less than 10 min	1,217			961			147			43			
10–30 min	951			753			166			74			
30–60 min	493			404			94			46			
1–3 hr	460			416			95			52			
3 hr or more	744			652			134			72			
Emotional closeness to the biological parent (i.e., partner of stepparent)								2.87	1.10		2.38	1.13	0.60

*SD* = standard deviation.

### Data Analysis

Our response variables were based on adult children’s answers on their support to their parents. For the analysis, we restructured the data into a long format so that each participant had a row for each of their (step)parent, up to 3 rows/parents per respondent. To analyze all response variables in the same model simultaneously, we applied a path analysis (Kline, 2016). The residual covariances among all the outcome variables were included in the model because these outcomes were all broad proxies of intergenerational support and were measured from the same parent-offspring dyads. We used the ordinal scale response variables as continuous ones, owing to their five-category scale (Rhemtulla et al., 2012). If any, the biases of the regression estimates and their standard errors (*SE*s) are expected to be negligible in large samples, as of this study (Li, 2021). As the data were clustered within the respondents (i.e., the data included multiple observations per respondent), the *SE*s of the estimates were corrected for clustering within the individuals. All response variables were treated as continuous and estimated with a robust maximum-likelihood estimation, correcting for the *SE*s using a sandwich estimator.

The variables measuring the parents’ age consisted of a relatively large number of missing values (see Table 2): the stepparents’ age was missing in approximately 43% of the cases, while the share of the missing values was only ~6% for the biological parents. This is possibly due to the respondents’ inadequate knowledge about the stepparents. To handle the missing data in the variables (see Table 2), we employed multiple imputations and followed the guidelines given by von Hippel (2020) for the number of imputed datasets. We imputed 30 datasets using multiple imputations by chained equations (White et al., 2011). The imputed sample included 8,949 observations. We calculated the predictive margins from the regression models. All statistical analyses were performed using the Stata 16 software.

## Results

### Comparison Between the Parents

First, we examined whether less support was directed toward the stepparents in comparison to the biological ones. Indeed, the biological parents were provided with more financial (predictive margin ± *SE*: 0.24 ± 0.01), practical (predictive margin ± *SE*: 1.15 ± 0.01), and emotional support (predictive margin ± *SE*: 1.63 ± 0.01) than the stepparents (financial assistance: 0.09 ± 0.01; practical help: 0.68 ± 0.03; emotional support 0.76 ± 0.03; results not shown in tables).

**Figure 1.**
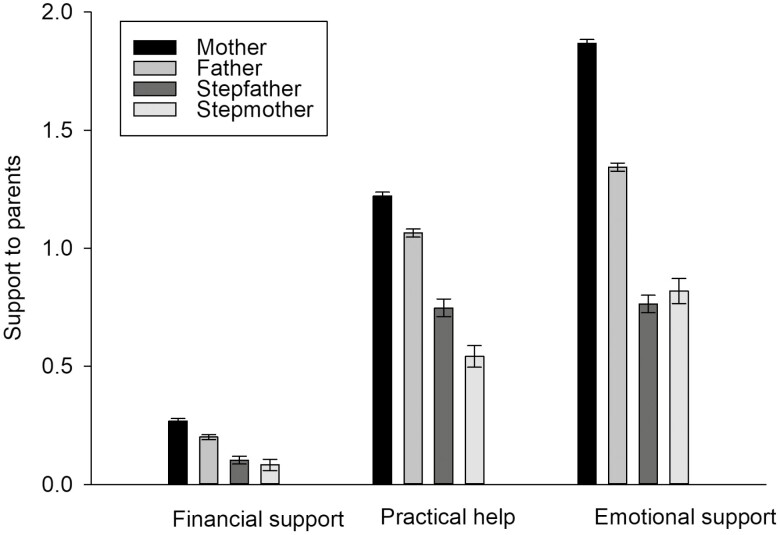
Support to parents and stepparents (predictive margins and standard errors; *N* = 8,949).

Subsequently, we investigated the support adult children provided to the different parent types. The results are presented in Table 3 and illustrated in Figure 1, which shows the predictive margins of the provided support. The stepmothers (predictive margin ± *SE*: 0.08 ± 0.02) as well as stepfathers (predictive margin ± *SE*: 0.10 ± 0.02) were provided less financial aid than the biological mothers (predictive margin ± *SE*: 0.27 ± 0.01) or fathers (predictive margin ± *SE*: 0.20 ± 0.01). Similarly, practical help was given less to the stepmothers (predictive margin ± *SE*: 0.54 ± 0.02) and stepfathers (predictive margin ± *SE*: 0.75 ± 0.04) in comparison to the biological mothers (predictive margin ± *SE*: 1.22 ± 0.02) or fathers (predictive margin ± *SE*: 1.06 ± 0.02). Finally, less emotional support was directed to the stepmothers (predictive margin ± *SE*: 0.82 ± 0.05) and stepfathers (predictive margin ± *SE*: 0.76 ± 0.04) compared to the biological mothers (predictive margin ± *SE*: 1.87 ± 0.02) or fathers (predictive margin ± *SE*: 1.34 ± 0.02). The above results also indicate that biological mothers were provided with more support in all three categories compared to biological fathers. In contrast, adult children gave more practical and financial help to stepfathers than to stepmothers, although stepmothers were directed slightly more emotional support than stepfathers.

**Table 3.** Results From Model Testing Relationship Between Parent Type and Different Investment Measures (*N* = 8,949)

Estimate	Coeff.	*SE*	*t*	*p*
Financial help to parents				
Parent (mother ref.)				
Father	−0.07	0.01	−6.39	<.0001
Stepfather	−0.17	0.02	−9.78	<.0001
Stepmother	−0.19	0.03	−7.31	<.0001
Gender				
Female	−0.06	0.02	−3.33	.001
Birth cohort (1981–1983 ref.)				
1971–1973	−0.02	0.03	−0.93	.353
Ethnic background (German native ref.)				
Other	0.23	0.03	9.12	<.0001
Level of education	−0.05	0.01	−5.12	<.0001
Co-habitation status (not co-habitating ref.)	−0.03	0.02	−1.66	.096
Parenthood status (no children ref.)	0.00	0.02	0.00	.998
Age of parent	0.00	0.00	0.48	.630
Travel time to parent’s home	0.00	0.01	−0.19	.850
Parental co-habitation (none ref.)	−0.18	0.02	−7.59	<.0001
Constant	0.57	0.09	6.31	<.0001
Practical help to parents				
Parent (mother ref.)				
Father	−0.16	0.02	−9.77	<.0001
Stepfather	−0.47	0.04	−13.07	<.0001
Stepmother	−0.68	0.05	−14.41	<.0001
Gender				
Female	−0.17	0.03	−5.85	<.0001
Birth cohort (1981–1983 ref.)				
1971–1973	−0.27	0.04	−6.75	<.0001
Ethnic background (German native ref.)				
Other	0.11	0.04	2.99	.003
Level of education	−0.03	0.01	−1.83	.070
Co-habitation status (not co-habitating ref.)	−0.13	0.03	−3.87	<.0001
Parenthood status (no children ref.)	−0.08	0.04	−2.35	.015
Age of parent	0.02	0.00	7.84	<.0001
Travel time to parent’s home	−0.26	0.01	−29.81	<.0001
Parental co-habitation (none ref.)	−0.11	0.03	−3.49	<.0001
Constant	1.45	0.14	10.42	<.0001
Emotional support to parents				
Parent (mother ref.)				
Father	−0.53	0.02	−28.63	<.0001
Stepfather	−1.10	0.04	−29.25	<.0001
Stepmother	−1.05	0.06	−18.85	<.0001
Gender				
Female	0.37	0.03	13.61	<.0001
Birth cohort (1981–1983 ref.)				
1971–1973	0.37	0.03	13.61	.171
Ethnic background (German native ref.)				
Other	0.17	0.04	4.68	<.0001
Level of education	0.06	0.01	3.98	<.0001
Co-habitation status (not co-habitating ref.)	0.05	0.03	1.43	.150
Parenthood status (no children ref.)	−0.14	0.03	−4.18	<.0001
Age of parent	0.01	0.00	3.15	.002
Travel time to parent’s home	−0.02	0.01	−2.06	.040
Parental co-habitation (none ref.)	−0.06	0.03	−1.90	.057
Constant	0.84	0.14	6.24	<.0001
var(e.financial help)	0.4	0.02		
var(e.practical help)	1.06	0.02		
var(e.emotional support)	1.05	0.02		
cov(e.financial help,e.practical help)	0.08	0.01	8.22	<.0001
cov(e.financial help,e.emotional support)	0.08	0.01	9.17	<.0001
cov(e.practical help,e.emotional support)	0.32	0.01	21.43	<.0001

*SE* = standard error.

In addition to the parent type, several other factors were associated with the provision of support to the stepparents. Females, respondents whose parents were born in Germany, and those whose parents were co-habiting with someone provided less financial support than males, respondents whose parents were not born in Germany, and those whose parents were living alone. Higher educational levels and longer travel times to the parents’ homes were associated with less financial assistance. Being female, belonging to an older cohort, not co-habiting, having children, having longer travel time to parents’ home, and co-habiting with parents were associated with less practical help. More practical help was given to those parents who were older and born outside Germany. More emotional support was provided by females, respondents whose parents were born outside Germany, highly educated respondents, and older parents. Less emotional support was related to having children and a longer distance from their parents.

### Childhood Co-residence Duration and Help to the Stepparents

**Figure 2.**
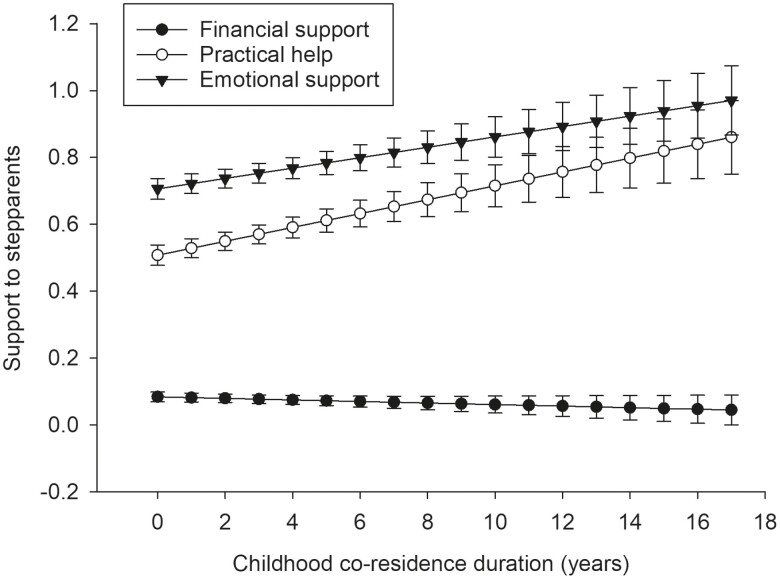
Support to the stepparents based on the childhood co-residence duration (predictive margins and standard errors; *N* = 984).

Next, we investigated whether the length of co-residence was associated with the support directed toward a stepparent (*N* = 984). These results are illustrated in Figure 2, and the statistical details of the full model are presented in Supplementary Table 1. We found that the childhood co-residence duration (years) was positively correlated with practical help and emotional support provided to the stepfathers and stepmothers. For example, the predictions (predictive margin ± *SE*) for providing practical help to the stepparents in 0, 5, and 17 years of co-residence were 0.51 ± 0.03, 0.61 ± 0.03, and 0.86 ± 0.10, respectively; and for emotional support, 0.71 ± 0.03, 0.78 ± 0.03, and 0.97 ± 0.10. However, we did not detect such an association regarding giving financial aid to the stepparents (0.08 ± 0.01, 0.07 ± 0.01, and 0.04 ± 0.05 for 0, 5, and 17 years, respectively).

These results suggest that a longer co-residence with the stepparents during childhood and adolescence reduces the step-gap. However, even in comparison to those who had co-resided with the stepparents for 17 years, still more practical help (predictive margin ± *SE*: 1.19 ± 0.02) and emotional support (predictive margin ± *SE*: 1.65 ± 0.02) was given to the biological parents with whom the respondents had lived their entire childhood (results not shown in tables); while a longer co-residence narrows the step-gap, it does not eliminate it.

## Conclusion

The current study investigated the step-gap in upward support by focusing on whether less support is provided to the stepparents than to the biological parents, and whether the length of co-residence during childhood and adolescence is associated with the provided support to the stepparents. It was found that adult children provided more financial aid, practical help, and emotional support to their biological parents than their stepparents. These findings align with previous studies on the step-gap phenomenon, which have shown that parents provide less *downward* support to their stepchildren than biological children (e.g., Arat et al., 2021; Pettay et al., 2023), adult children perceive weaker *normative obligations* to support their stepparents as opposed to their biological parents (van Houdt et al., 2018), and the *relationship quality* between stepparents and stepchildren is generally lower when compared to the relationships between biological parents and their children (e.g., Arránz Becker et al., 2013; Steinbach & Hank, 2016). However, this study is the first to provide evidence regarding the existence of step-gap in upward support, relying on a population-based sample that includes parents from diverse sociodemographic groups (cf. Pezzin et al., 2008).

Our results are in line with the “biological relatedness effect” and the “social norms effect,” both suggesting that people tend to prefer their biological parents over stepparents. Although these two concepts are based on different theoretical viewpoints, it is important to note that the perspectives are not necessarily contradictory; instead, they can rather complement each other. Initially, individuals can be inclined to offer more support to their biological parents than to their stepparents. Over time, though, social norms may evolve, leading to increased expectations and obligations to support stepparents and thus narrow the width of the step-gap in upward support.

Following this notion, we explored the dynamics of upward support further and discovered that when adult children had co-resided longer with their stepparents during childhood, they offered more practical help and emotional support to their stepparents. These findings provided evidence for the “childhood co-residence effect” and align with previous research on *downward* support, which has consistently shown that a longer period of co-residence between stepparents and stepchildren is linked to increased support from stepparents (Kalmijn, 2013; Kalmijn et al., 2019; Pettay et al., 2023). The results suggest that due to the prolonged co-residence with a stepparent, the stepchild–stepparent bond may become more kin-like in terms of the provided support. However, we did not detect an association between co-residence and the provision of financial aid, which may be related to the fact that financial support typically flows downward between the family generations, and adult children rarely provide any financial aid to older generations (e.g., Hämäläinen & Tanskanen, 2021).

Moreover, we found that adult children provided more practical, emotional, and financial support to their biological mothers than to their fathers. This pattern might be linked to the fact that, due to sociocultural, psychological, and biological reasons, mothers tend to take on the role of primary parents and, on average, invest more time and resources in their children during childhood compared to fathers (e.g., Hrdy, 2009). Consequently, due to a long-term reciprocity effect, adult children may provide more support toward their mothers than their fathers later in life.

Regarding step-relationships, we observed that while slightly more emotional support was provided to stepmothers than stepfathers, financial assistance and practical help were directed more toward stepfathers than stepmothers. This outcome may reflect the typical living arrangements following parental separation in Germany, where children usually reside with their mothers rather than their fathers (Walper et al., 2021). Such living arrangements can foster closer bonds with mothers’ new partners (stepfathers) as opposed to fathers’ new partners (stepmothers), and also increase the social expectations to channel more support toward stepfathers than stepmothers in later stages of life. Furthermore, stepfathers may often receive support from stepchildren because they are the partners of the primary family kin keeper, the biological mother. However, stepmothers lack this same connection to primary kin keepers who can bridge the gap between them and their partners’ children. As a result, the bond between stepmothers and stepchildren may not be as strong as that between stepfathers and stepchildren.

Our study has several strengths. We employed a rigorous analytical approach and utilized population-based data to examine multiple measures of intergenerational support directed toward four types of parents separately (i.e., mothers, fathers, stepmothers, and stepfathers); this allowed us to conduct a comprehensive analysis of the upward step-gap. Moreover, we examined whether the childhood co-residence duration affected upward support and therefore the width of the step-gap, which had not been previously investigated.

Our research has some limitations. Owing to the insufficient data on stepmothers, the patterns between the childhood co-residence duration and help given to the stepparents were likely driven by the stepfathers. Due to the inadequate variation in the childhood co-residence duration with the stepmothers, we could not investigate whether the relationship between the co-residence duration and helping stepparents differs between the stepmothers and stepfathers. While we argue that the length of childhood co-residence is likely to influence the width of the step-gap in upward support, our data did not allow us to draw definite causal conclusions. To further examine the more causal associations, future research could assess the relationship between co-residence and the step-gap by using the data that includes multiple children from the same families, as well as examine whether the provision of upward support varies among siblings according to the length of co-residence with their stepparents. Furthermore, future research could benefit from considering the presence of half- and step-siblings, which may affect the provision of intergenerational support. For instance, stepchildren may provide less support if their stepparents have biological children; conversely, parents with both biological and stepchildren can potentially receive support from multiple sources, thereby diluting the negative effects of the step-gap. Moreover, the parents of this study’s respondents were still relatively young (mean ages between 56 and 61 years), and they might not have had a compelling need to receive support from their children thus far. Future studies should examine whether the step-gap is associated with parental needs, and consider factors such as parents’ health or socioeconomic status. Finally, our results were based on a sample of German individuals which may limit their generalizability to other countries. Future research should explore whether a step-gap also exists in other countries and whether it differs between diverse cultural settings.

Our findings have important implications for understanding the dynamics of intergenerational relationships among stepfamilies. They suggest that the stepparents are in a more disadvantaged position than the biological parents regarding receiving support from their adult children. As the share of stepfamilies increases (Thomson, 2014) and the old-age dependency ratio worsens (OECD, 2023), the step-gap may have significant consequences for stepparents, particularly later in life, when individuals typically need more support from their families due to declining functional abilities or health problems. Indeed, a prior study found that older stepmothers were institutionalized sooner and stepfathers deceased “younger” compared to the older parents with biological children (Pezzin et al., 2013); this possibly indicates that stepparents may not have the possibility to receive adequate support from their families. Regarding aging societies, it is crucial to acknowledge the challenges faced by the aging individuals with their stepfamilies and explore how to reduce the negative effects of the step-gap on the well-being of older individuals.

## Supplementary Material

gbad179_suppl_Supplementary_Tables_S1
