# Supplementary material for: Step-Gap in Upward Support: The Role of Biological Relatedness and Childhood Co-residence Duration
Source: J Gerontol B Psychol Sci Soc Sci. 2023 Dec 21;79(4):gbad179. doi: 10.1093/geronb/gbad179 (PMC10944143; doi:10.1093/geronb/gbad179)
Supplement: gbad179_suppl_Supplementary_Tables_S1 [file gbad179_suppl_supplementary_tables_s1.docx]

**Online Supplementary Material**

Supplementary Table 1. Results on regression investigating childhood co-residence duration and help to stepparents (N=984).

| **Estimate** | | **coeff.** | **SE** | **t** | **p** |
| --- | --- | --- | --- | --- | --- |
| *Financial help to parents* | |  |  |  |  |
| Parent (stepfather ref.) | |  |  |  |  |
|  | Stepmother | 0.00 | 0.03 | 0.14 | 0.887 |
| Childhood co-residence duration | | 0.00 | 0.00 | -0.77 | 0.440 |
| Gender | |  |  |  |  |
|  | Female | -0.03 | 0.03 | -1.24 | 0.215 |
| Birth cohort (1981-1983 ref.) | |  |  |  |  |
|  | 1971-1973 | -0.04 | 0.04 | -0.96 | 0.339 |
| Ethnic background (German native ref.) | |  |  |  |  |
|  | Other | 0.07 | 0.04 | 1.70 | 0.090 |
| Level of education | | -0.02 | 0.01 | -1.14 | 0.255 |
| Co-habitation (no co-habition ref.) | | 0.01 | 0.03 | 0.32 | 0.753 |
| Parenthood status (no children ref.) | | 0.05 | 0.03 | 1.91 | 0.056 |
| Age of parent | | 0.00 | 0.00 | -0.03 | 0.977 |
| Travel time to parent's home | | -0.02 | 0.01 | -1.75 | 0.080 |
| Parental co-habitation (none ref.) | | 0.07 | 0.02 | 3.55 | <.0001 |
| Emotional closeness to biological parent | | 0.03 | 0.01 | 3.08 | 0.002 |
| Constant | | 0.06 | 0.13 | 0.45 | 0.655 |
| *Practical help to parents* | |  |  |  |  |
| Parent (stepfather ref.) | |  |  |  |  |
|  | Stepmother | -0.13 | 0.06 | -2.26 | 0.024 |
| Childhood co-residence duration | | 0.02 | 0.01 | 2.94 | 0.003 |
| Gender | |  |  |  |  |
|  | Female | -0.21 | 0.06 | -3.52 | <.0001 |
| Birth cohort (1981-1983 ref.) | |  |  |  |  |
|  | 1971-1973 | -0.12 | 0.07 | -1.68 | 0.093 |
| Ethnic background (German native ref.) | |  |  |  |  |
|  | Other | 0.06 | 0.08 | 0.79 | 0.431 |
| Level of education | | -0.03 | 0.03 | -0.96 | 0.339 |
| Co-habitation (no co-habition ref.) | | -0.09 | 0.07 | -1.33 | 0.185 |
| Parenthood status (no children ref.) | | 0.03 | 0.07 | 0.48 | 0.631 |
| Age of parent | | 0.01 | 0.00 | 1.53 | 0.126 |
| Travel time to parent's home | | -0.11 | 0.02 | -5.75 | <.0001 |
| Parental co-habitation (none ref.) | | 0.24 | 0.06 | 3.71 | <.0001 |
| Emotional closeness to biological parent | | 0.15 | 0.02 | 6.31 | <.0001 |
| Constant | | 0.36 | 0.27 | 1.36 | 0.174 |
| *Emotional support to parents* | |  |  |  |  |
| Parent (stepfather ref.) | |  |  |  |  |
|  | Stepmother | 0.21 | 0.06 | 3.29 | 0.001 |
| Childhood co-residence duration | | 0.02 | 0.01 | 2.34 | 0.019 |
| Gender | |  |  |  |  |
|  | Female | 0.21 | 0.06 | 3.57 | <.0001 |
| Birth cohort (1981-1983 ref.) | |  |  |  |  |
|  | 1971-1973 | 0.09 | 0.08 | 1.15 | 0.250 |
| Ethnic background (German native ref.) | |  |  |  |  |
|  | Other | 0.08 | 0.09 | 0.90 | 0.368 |
| Level of education | | 0.00 | 0.03 | 0.00 | 1.000 |
| Co-habitation (no co-habition ref.) | | 0.12 | 0.07 | 1.81 | 0.071 |
| Parenthood status (no children ref.) | | -0.19 | 0.08 | -2.48 | 0.013 |
| Age of parent | | 0.00 | 0.00 | 0.35 | 0.729 |
| Travel time to parent's home | | 0.02 | 0.02 | 0.95 | 0.340 |
| Parental co-habitation (none ref.) | | 0.40 | 0.07 | 5.77 | <.0001 |
| Emotional closeness to biological parent | | 0.26 | 0.02 | 10.69 | <.0001 |
| Constant | | -0.90 | 0.27 | -3.33 | 0.001 |
| var(e.financial help) | | 0.16 | 0.03 |  |  |
| var(e.practical help) | | 0.74 | 0.04 |  |  |
| var(e.emotional support) | | 0.79 | 0.04 |  |  |
| cov(e.financial help,e.practical help) | | 0.04 | 0.02 | 2.63 | 0.009 |
| cov(e.financial help,e.emotional support) | | 0.04 | 0.02 | 2.54 | 0.011 |
| cov(e.practical help,e.emotional support) | | 0.25 | 0.03 | 7.81 | <.0001 |
